# J. H. Reed & Co.

**Published:** 1860-09

**Authors:** 


☞ Messrs. J. H. Reed & Co., whose card will be found on the cover of this journal, have recently fitted up rooms over their store, exclusively for their surgical and dental instruments. They have been compelled to make this change owing to the increase of trade in these goods, as also the necessity of more room to exhibit properly their large stock of instruments. They keep everything needed by the profession and have now a better arrangement for showing new materials as received. They request us to say that they shall always endeavor to keep all improvements and new instruments, and will be pleased to show their stock whether parties wish to purchase or not.

"The Medical Department of Baker University," is the name of a new school announced as established at Leavenworth, City, K. T.

### ITEMS.

Dr. J. E. Holbrook has resigned the Chair of Anatomy in the Medical College of the State of South Carolina.

A fifth medical school has been organized at Griffin, in the State of Georgia, under the name of the Middle Georgia Medical School.

The proceedings of the Sixty-eighth Annual Convention of the Connecticut Medical Society, indicate the condition of the profession in that State to be vital and progressive.

G. M. B. Maughs, M. D., senior editor of the *Kansas City Medical and Surgical Review*, has accepted the appointment of Professor of Physiology and Chemistry in the Medical Department of the Missouri State University, at St. Louis.

The *Cleveland Medical Gazette* and the *Cincinnati Lancet and Observer*, have, in effect, been merged in one; containing, as they do, the same matter, though retaining their original names, and purporting to be issued from different cities.

